# Hospital Promotion

**Published:** 1886-11-20

**Authors:** 


### Hospital Promotion: A Wag's Experience.

The Editor's Explanation.

A young medical practitioner of promise, having satisfied the examiners and obtained his diplomas, sat down to consider what steps he should take as best calculated to lead him to fortune. He had held all the resident appointments (H. S., H. P., and R. O.), and was a man of parts and enterprise. In the result he advertised in the papers for a suitable opening for his energies. Naturally, he preferred to remain in London and to aspire to the higher walks of the profession, and these views were duly noted in his advertisement One response promised him much that he desired, but the terms were so remarkable, and the plan proposed so original, as to merit permanent preservation in these columns. We will now let him tell his experiences in his own words, only premising that the story is so realistic as to cause some doubt whether this young practitioner is speaking the actual truth, or is only desirous of perpetrating a joke before he settles down to the sobering influences of every-day practice. This question our readers must settle for themselves.

What the Advertisement produced.

"Many answers came to my advertisement," says he, a fact due, no doubt, to the circumstance that I incidentally mentioned I had at my disposal some £2,000. The one which possessed most attractions for me was a neat little note signed Jeremiah Diddler, who requested the favour of an interview, which he felt sure would lead to business. Mr. Diddler accordingly called upon me one morning, and we had a prolonged consultation, which resulted in his placing the following proposals before me. Apart from their merits, I could not fail to note the frank and reassuring manners of Mr. Diddler, and his evident desire to prove himself an agreeable companion as well as an honest man.

Mr. Diddler proposes.

The great man explained that he had for twenty years devoted himself to the business of "hospital promotion," and that his methods were at once simple and successful. He had at the present time a scheme for the establishment of a new Special Hospital that could not fail to meet a felt want and to make the fortune of any medical man who associated himself with it as founder. Mr. Diddler was prepared with the names of a duke, a right honourable, and a bishop, with those of other more or less distinguished personages whose patronage he would guarantee to secure, together with a treasurer, bankers, and a committee of management, consisting of people whose position and standing were unquestionably good. He further explained that it would be necessary to have a list of subscribers and donors to act as decoy-ducks. These names would not be so difficult to secure as might at first be imagined. For instance, Mr. Diddler has at his disposal, for a consideration, the services of a man who has devoted his efforts for years to the collection of accurate lists of persons who are specially willing to contribute to the particular class of institution for which the appeal is to be made. It may seem remarkable, said Mr. Diddler, but it is an undoubted fact, that most benevolent people who contribute liberally to charities have a special leaning towards one distinct group. The gentleman referred to has arranged the names in classes. Class No. 1 consists of those whose sympathies are roused with the greatest ease. From this list he selects the more susceptible, and by writing several autograph letters secures in a few weeks all the names he requires for his provisional list. A circular, once printed, will serve for an indefinite period; for it is noticeable, as an evidence of the truth of this fact, that Mr. Diddler finds it to be desirable that few, if any, of this class of appeals shall bear a date of issue. The process of raising money thus becomes almost mechanical. It consists in sending forth a number of appeals accompanied by a letter duly elaborated and enclosed in an irreproachable envelope. If the victim will not rise to the first cast, a judicious reminder, repeated at intervals of about three months, recalls his or her attention to the pressing needs and undoubted claims of the promoter's noble and excellent institution. Success is practically certain under this system, but even then, as Mr. Diddler explains, his resources are not exhausted, for his class of business has so increased of late years, that it now possesses a literature of its own, including a newspaper with an appropriate title. This independent organ of public opinion is willing to insert any article which Mr. Diddler may write, in return for which the latter purchases some 500 copies of the number containing the article, and circulates them among the persons whom he deems likely to read the production and to be sympathetically moved by it. Mr. Diddler proudly produces a copy of the *Looking Glass*, for so the paper is called, and it is only fair to state that many of the articles are compiled with great force and ability.

Mr. Diddler's Appeal.

Having made this statement by way of introduction, the ingenious promoter brings forth the following circular letter of appeal, with the explanation that his nephew has been in full swing for two years over a baker's shop in a crowded district of London, and the statements in the appeal are therefore absolutely reliable. He will not, however, expect any compensation for the goodwill of the dispensary, though he will hand it over as a going concern.

FREE DISPENSARY FOR DISEASES OF THE LITTLE TOE.

—— Street, —— Square.

In the year 1884, 30,807 persons died of diseases of the little toe. This number (which includes cases of gangrene, pyæmia, phlebitis, and bunion) is exclusive of the very numerous deaths from wasting diseases and cancer of the digit. (See *Registrar-General's Report*.)

According to the above extract, diseases of the little toe have occasioned more deaths in England than diseases of any other organ, and a comparison of the returns for 1883 with those of previous years shows that these diseases are increasing in a ratio greatly out of proportion to the increasing population.

*Deaths from Diseases of the Little Toe.*

| Year. | | Year. | | Year. | |
|---|---|---|---|---|---|
| 1874 | 13,846 | 1877 | 17,908 | 1880 | 21,915 |
| 1875 | 15,401 | 1878 | 19,634 | 1881 | 30,940 |
| 1876 | 15,009 | 1879 | 20,879 | 1882 | 24,802 |
| 1883 | 30,807 | | | | |

Besides the above number of fatal cases there is a still greater number of diseases of the little toe not terminating in death, but which give rise to extreme suffering and misery, especially amongst the poorer classes. *This is the only institution specially devoted to the treatment of diseases of the little toe*, and the fact that in less than two years upwards of 10,560 patients (from all parts of England) have been relieved, proves that it is a necessity. The employment of the newly-invented instrument (the little-toe scalpel) is a special feature in the institution, and by the aid of this instrument morbid conditions of the digit can be fully exposed and adequately treated. Further aid is required to meet the urgent demands caused by the daily increasing number of out-patients. It is likewise essential that a ward should be speedily opened for the reception of the more acute cases. *At least £1,000 is required to establish a ward containing six beds.* For this purpose the committee have determined to open a separate subscription list. Those who desire to assist in this particular way, will please notify that their subscriptions or donations are to go to the "Ward Fund." The benevolent are respectfully informed that the very existence of the institution will be jeopardised unless the funds are considerably augmented.

JEREMIAH DIDDLER, Secretary.

Attached to this circular were the names of the duke, the right honourable, and the bishop aforesaid, together with those of other more or less distinguished personages, and the committee of management, treasurer, and bankers all complete. "There!" said Mr. Diddler, when I had read it, his face beaming with satisfaction, "you agree to found this hospital under my auspices, and you will one day be a baronet, at least, without the shadow of a doubt."

An answer anticipated.

Such are Mr. Diddler's proposals, and I am to have a month to consider them. Can you let me know if there is any law or reason why we should not proceed to found this hospital on Mr. Diddler's system, if we consider it desirable in our own interests or for any other reason to do so?

[Perhaps some one will answer this question, which strikes us as rather a poser?—ED.]

